# Posicionamento sobre Uso de Antiplaquetários e Anticoagulantes nos Pacientes Infectados pelo Novo Coronavírus (COVID-19) – 2020

**DOI:** 10.36660/abc.20200424

**Published:** 2020-08-19

**Authors:** Alexandre de Matos Soeiro, Tatiana de Carvalho Andreucci Torres Leal, Marcel de Paula Pereira, Eduardo Gomes Lima, Ana Cristina Baptista da Silva Figueiredo, João Luiz Fernandes Petriz, Dalton Betolim Precoma, Carlos Vicente Serrano

**Affiliations:** 1 Hospital das Clínicas Faculdade de Medicina Universidade de São Paulo São Paulo SP Brasil Instituto do Coração (Incor), Hospital das Clínicas da Faculdade de Medicina da Universidade de São Paulo (HCFMUSP),São Paulo, SP – Brasil; 2 Hospital BP Mirante São Paulo SP Brasil Hospital BP Mirante, São Paulo, SP – Brasil; 3 Hospital 9 de Julho São Paulo SP Brasil Hospital 9 de Julho, São Paulo, SP – Brasil; 4 Hospital Glória D’Or Rio de Janeiro RJ Brasil Hospital Glória D’Or, Rio de Janeiro, RJ – Brasil; 5 Hospital Barra D’Or Rio de Janeiro RJ Brasil Hospital Barra D’Or, Rio de Janeiro, RJ – Brasil; 6 Sociedade Hospitalar Angelina Caron Curitiba PR Brasil Sociedade Hospitalar Angelina Caron, Curitiba, PR – Brasil; 7 Pontifícia Universidade Católica do Paraná Curitiba PR Brasil Pontifícia Universidade Católica do Paraná, Curitiba, PR – Brasil

**Table:** 

Declaração de potencial conflito de interesses dos autores/colaboradores do Posicionamento do Departamento de Ergometria, Exercício, Cardiologia Nuclear e Reabilitação Cardiovascular (DERC/SBC) sobre a Atuação Médica em suas Áreas Durante a Pandemia por COVID-19 Se nos últimos 3 anos o autor/colaborador do Posicionamento:							
Nomes Integrantes do Posicionamento	Participou de estudos clínicos e/ou experimentais subvencionados pela indústria farmacêutica ou de equipamentos relacionados ao posicionamento em questão	Foi palestrante em eventos ou atividades patrocinadas pela indústria relacionados ao posicionamento em questão	Foi (é) membro do conselho consultivo ou diretivo da indústria farmacêutica ou de equipamentos	Participou de comitês normativos de estudos científicos patrocinados pela indústria	Recebeu auxílio pessoal ou institucional da indústria	Elaborou textos científicos em periódicos patrocinados pela indústria	Tem ações da indústria
Antônio Carlos Avanza Jr.	Não	Não	Não	Não	Não	Não	Não
Carlos Alberto Cordeiro Hossri	Não	Não	Não	Não	Não	Não	Não
Carlos Alberto Cyrillo Sellera	Não	Não	Não	Não	Não	Não	Não
Gabriel Blacher Grossman	Não	Não	Não	Não	Não	Não	Não
Lara Terra F. Carreira	Não	Não	Não	Não	Não	Não	Não
Luiz Eduardo Fonteles Ritt	Não	Não	Não	Não	Não	Não	Não
Luiz Eduardo Mastrocola	Não	Não	Não	Não	Não	Não	Não
Mauricio Batista Nunes	Não	Não	Não	Não	Não	Não	Não
Mauricio Milani	Não	Não	Não	Não	Não	Não	Não
Nabil Ghorayeb	Não	Não	Não	Não	Não	Não	Não
Odilon Gariglio Alvarenga de Freitas	Não	Não	Não	Não	Não	Não	Não
Pedro Ferreira de Albuquerque	Não	Não	Não	Não	Não	Não	Não
Romeu Sergio Meneghelo	Não	Não	Não	Não	Não	Não	Não
Salvador Manoel Serra	Não	Não	Não	Não	Não	Não	Não
Tales de Carvalho	Não	Não	Não	Não	Não	Não	Não
William Azem Chalela	Não	Não	Não	Não	Não	Não	Não

## 1. Introdução

A pandemia pelo novo coronavírus (SARS-CoV-2) vem gerando debates a respeito do melhor tratamento para a doença e suas complicações. Publicações recentes demonstraram que as doenças cardiovasculares (DCV) estão entre os principais fatores de risco para evolução desfavorável da doença, incluindo hipertensão arterial e diabetes mellitus.^1 - 6^

Foi demonstrado que pacientes com infecção pelo novo coronavírus (COVID-19) apresentam mecanismos pró-trombóticos distintamente ativados, com maior possibilidade de eventos trombóticos ocorrerem. Síndrome coronariana aguda (SCA) com e sem supradesnivelamento do segmento ST pode ocorrer em pacientes com COVID-19, mas a real incidência ainda é incerta.^7 - 10^

Dessa forma, diversas questões relacionadas ao uso de medicamentos antiplaquetários e anticoagulantes em pacientes com suspeita ou infecção confirmada por COVID-19 permanecem incertas. Tais recomendações a seguir são válidas para as mais diversas situações clínicas como fibrilação atrial, síndrome coronariana aguda, doença arterial coronariana crônica, intervenção coronariana percutânea, pós-cirurgia cardíaca, acidente vascular encefálico (AVE) isquêmico e tromboembolismo venoso, devendo ser aplicadas caso-a-caso.

## 2. Fisiopatologia Envolvida

### 2.1. Mecanismo de Entrada Celular

O vírus SARS-CoV-2 tem como receptor funcional e porta de entrada a enzima conversora da angiotensina 2 (ECA2). Trata-se de uma carboxipeptidase que, diferente da enzima conversora da angiotensina 1, tem efeito contrário, aumentando a degradação da angiotensina 2 e, portanto, apresenta efeito final vasodilatador. Além de estar presente no parênquima pulmonar, a ECA2 é distribuída também em todo sistema cardiovascular, rins e coração. Sabe-se que a ECA2 tem certa participação na função ventricular. Modelos animais que tem expressão reduzida de ECA2, apresentam disfunção ventricular esquerda severa. Aparentemente a infecção pelo novo coronavírus é capaz de promover *downregulation* desses receptores, o que poderia favorecer a injúria miocárdica e a lesão pulmonar.^1 , 2 , 11^ . Apesar dessa possível associação, estudo observacional que avaliou 8910 pacientes infectados pelo SARS-Cov-2 não demonstrou aumento de mortalidade em pacientes em uso de medicações inibidores da ECA (IECA) e bloqueador de receptores de angiotensina (BRA).^12^ Desta forma, essas medicações devem ser mantidas em pacientes que faziam uso prévio e desenvolverem a infecção.

### 2.2. Injúria Miocárdica

Diversos estudos, principalmente chineses, vêm demonstrando o impacto da injúria miocárdica na instalação e progressão da COVID-19, assim como em sua apresentação de gravidade. Dados chineses descreveram a presença de injúria miocárdica em aproximadamente 20-30% dos pacientes internados, sendo presente também em 40% naqueles que morreram. Há dados demonstrando que a elevações de alguns marcadores, como troponina e D-dímero, estão associados a pior prognóstico, com maior necessidade de internação em unidades de terapia intensiva, necessidade de ventilação mecânica e morte.^2 , 13 , 14^

O mecanismo da injúria miocárdica ainda não está bem estabelecido. Além do efeito direto do vírus, há o envolvimento do estresse miocárdico induzido pela falência respiratória e hipoxemia, com desbalanço de oferta e demanda, além de uma ação indireta da resposta inflamatória sistêmica sobre o tecido miocárdico e função endotelial.^14 - 19^

Não há dados suficientes sobre o efeito da inflamação miocárdica. Não se sabe ao certo se a miocardite induzida pela COVID-19 produza insuficiência cardíaca com fração de ejeção exclusivamente reduzida. Há relatos que descrevem achados anatomopatológicos de miocardite linfocítica, mesmo em pacientes com fração de ejeção preservada e sinais de hipertrofia ventricular.^14 - 19^

### 2.3. Associação com SCA

O risco aumentado para SCA em portadores da doença pode ser explicado pelo aumento da atividade trombótica descrita nesses pacientes, evidenciado pela frequente elevação do D-dímero e plaquetopenia. Além desse achado específico, sabe-se que há um aumento de eventos coronarianos em associação direta de infecções respiratórias virais. Fatores relacionados à atividade inflamatória, tais como disfunção endotelial, ativação plaquetária, ativação de macrófagos, disfunção hepática, expressão de fatores teciduais e liberação de citocinas são capazes de aumentar o risco de instabilização da placa aterosclerótica. Estudo mais recentes identificaram também níveis elevados de anticorpos antifosfolípides em pacientes com COVID-19, no entanto, não se sabe se isso apresenta alguma relação com gravidade da doença.^5 , 7 - 10 , 15 , 20^

No início da fase de infecção, a inibição plaquetária pode reduzir a formação de fibrina intravascular e trombos. Dessa forma, uso de aspirina pré-hospitalar, mas não o uso pós-admissão, foi associado menor risco para o desenvolvimento de insuficiência respiratória e mortalidade em pacientes com pneumonia adquirida pela comunidade. Em segundo lugar, a escolha de inibidores P2Y12 orais: apesar do fato de que todos os inibidores P2Y12 reduzem agregados de plaquetas e leucócitos e citocinas pró-inflamatórias derivadas de plaquetas, ticagrelor é único em ter um alvo adicional bem documentado de inibição, transportador nucleosídeo equilibrativo 1 (ENT1), contribuindo para a inibição da captação de adenosina celular. Portanto, ticagrelor confere propriedades anti-inflamatórias mais potentes, embora não testado em COVID-19.^7^

Vale ressaltar que na vigência do quadro clínico infeccioso e, visto a alta incidência de injúria miocárdica decorrente a presença direta do vírus e de efeitos indiretos da infecção, mesmo na vigência de alterações eletrocardiográficas, o diagnóstico de SCA deve ser considerado frente à possibilidade de doença não isquêmica. Foi publicado^21^ relato de 18 casos de elevação do segmento ST em hospitais de Nova York, sugerindo SCA com supra de ST. Dentre eles, 4 tinham elevação difusa e 14 tinham elevações focais do segmento ST. 50% dos pacientes fizeram cineangiocoronariografia e 33% não tinham doença coronariana obstrutiva. No total, 44% dos pacientes tiveram diagnóstico de infarto agudo do miocárdio. Dessa forma, mesmo em pacientes com elevação do segmento de ST, diagnósticos diferenciais devem ser levantados, visto quadro clínico heterogêneo. Independente da etiologia, a mortalidade foi de 72%. A decisão terapêutica e a estratificação invasiva deve ser ponderada levando em consideração quadro clínico, achados de exames complementares, experiência da equipe e disponibilidade do laboratório de hemodinâmica.^14 , 16^ . Em outras duas publicações, demonstraram-se que houve menor incidência de SCA no norte da Califórnia, em relação ao mesmo período no ano anterior e que houve maior incidência de parada cardíaca extra-hospitalar, na Itália. Isto sugere uma menor procura da população aos serviços de emergência.^22 , 23^

Além de todos esses fatores, uma enorme preocupação tem sido levantada devido aos efeitos do isolamento social. A maioria das pessoas reduziram drasticamente a atividade física. Além disso, a alimentação por vezes torna-se inadequada com ingesta maior de carboidratos. Tais mudanças no estilo de vida podem ser fatores adicionais capazes de desencadear e contribuir com eventos trombóticos com AVE e SCA.^24^

### 2.4. Mecanismo Tromboembólico

Pacientes infectados pelo COVID-19 provavelmente apresentam risco aumentado de tromboembolismo venoso (TEV). Embora não haja nenhuma grande série de casos publicados até agora, há relatos de parâmetros anormais de coagulação em pacientes hospitalizados com doença COVID-19 grave.^25 - 27^

Estudo recente mostrou em uma série de 106 casos de COVID-19 submetidos à angiotomografia de artérias pulmonares na investigação de TEV pulmonar que 30% dos pacientes houve confirmação de tromboembolismo venoso. Pacientes com infecção por COVID-19 e embolia pulmonar apresentaram níveis mais elevados de D-dímero do que aqueles sem embolia ( *p* < 0,001), além de maior necessidade de internação em terapia intensiva (75% vs. 32%, p < 0,001). A presença de D-dímero > 2.660 µg/L apresentou sensibilidade de 100% e especificidade de 67% para embolia pulmonar.^28^

O D-dímero tem sido associado a maior taxa de mortalidade e parece aumentar progressivamente com a exacerbação da infecção. A fase da doença em que ocorre o desenvolvimento de síndrome do desconforto respiratório agudo (SDRA) e a piora do padrão radiológico é marcada pela elevação expressiva de D-dímero, observando-se nos casos mais graves injúria miocárdica e coagulação intravascular disseminada (CIVD). A resposta inflamatória sistêmica em pacientes com infecção pode resultar em lesão endotelial com consequente aumento na geração de trombina e redução da fibrinólise endógena. Esse estado pró-trombótico é denominado coagulopatia induzida pela sepse (SIC) e precede a CIVD. Os diversos mecanismos envolvidos na SIC agem simultaneamente, culminando em um estado pró-hemostático. Aparentemente, os fatores mais importantes que medeiam esse distúrbio do sistema de coagulação durante a sepse são as citocinas inflamatórias.^29^

Evidências demonstram uma interação cruzada entre inflamação e coagulação, com a inflamação induzindo a ativação da coagulação e a coagulação acentuando a atividade inflamatória. As plaquetas têm um papel central no desenvolvimento das anormalidades da coagulação na sepse e podem ser ativadas diretamente por mediadores pró-inflamatórios, como o fator ativador de plaquetas, bem como por meio da trombina gerada. A ativação de plaquetas também pode estimular a formação de fibrina por mecanismo alternativo. A expressão de P-selectina na membrana plaquetária não apenas induz a adesão de plaquetas a leucócitos e células endoteliais, mas também aumenta a expressão do fator tecidual nos monócitos. Em circunstâncias normais, a ativação da coagulação é controlada por três importantes vias anticoagulantes fisiológicas: o sistema antitrombina, o sistema ativado da proteína C e o inibidor da via do fator tecidual. Na sepse, todas as três vias sofrem disfunção. Em meio a todo esse desbalanço do sistema de coagulação, a fibrinólise endógena é amplamente reduzida.^29^

Em um estudo de coorte retrospectiva da China, níveis elevados de D-dímero (>1g/L) foram fortemente associados ao óbito hospitalar. Em outro estudo comparando sobreviventes da COVID-19 com não-sobreviventes, os não sobreviventes tiveram níveis significativamente mais elevados de D-dímero e produtos de degradação de fibrinas e 71,4% dos não sobreviventes atenderam aos critérios clínicos de CIVD durante o curso da doença. Além da CIVD, pacientes gravemente doentes com imobilização prolongada estão inerentemente em alto risco para TEV. A inflamação vascular também pode contribuir para o estado hipercoagulante e endotelial disfunção em tais pacientes. No cenário de pacientes COVID-19 gravemente doentes que demonstram clínico deterioração como evidenciado por hipóxia ou instabilidade hemodinâmica, doença tromboembólica deve ser considerada.^25 - 27^

## 3. Interações Medicamentosas e Efeitos Cardiovasculares Pró/Antitrombóticos

Até o momento, não há tratamento específico para a infecção pelo COVID-19 e um arsenal terapêutico vem sendo usado em situações de gravidade em ambiente hospitalar, muitos ainda em investigação de eficácia e segurança.

Como esses medicamentos podem ser usados em situações específicas, vale considerar seus efeitos colaterais sobre o sistema cardiovascular e possíveis interações medicamentosas com outras terapias frequentemente utilizadas em pacientes cardiopatas.

### 3.1. Antirretrovirais

A ribavirina, e o remdesivir são agentes que agem bloqueando a RNA polimerase e o lopinavir/ritonavir inibem a replicação viral.

Não há descrição de cardiotoxicidade induzido pela ribavirina. Por sua vez, o lopinavir/ritonavir produz alargamento do intervalo QT e PR, principalmente em pacientes que já apresentam QT longo ou estão em uso de outras drogas que também interagem sobre o intervalo QT. Tanto a ribavirina quanto o lopinavir/ritonavir potencializam o efeito anticoagulante, modificando a ação da varfarina (principalmente ribavirina) ou novos anticoagulantes como apixabana e rivaroxabana (principalmente lopinavir-ritonavir).^30 - 32^

Em outro estudo, o uso de dabigatrana em pacientes internados por COVID-19 com uso de antivirais, teve aumento dos níveis séricos plasmáticos, com necessidade de retirada da droga em mais da metade dos pacientes.^31^

O lopinavir-ritonavir podem também influenciar na atividade dos inibidores P2Y12 por inibição da CYP3A4 o que reduz o nível sérico de metabólitos ativos do clopidogrel e aumenta a atividade do ticagrelor. Assim, pelo alto risco de sangramento, o uso concomitante de ticagrelor e lopinavir-ritonavir deve ser desencorajado.^11^

Há também evidência de que o uso de clopidogrel na vigência de tratamento com lopinavir-ritonavir possa produzir efeito antiagregante insuficiente, o que não é observado com prasugrel, sendo, portanto, a droga mais ideal ^32^ Na presença de contraindicações para o uso de prasugrel (tais como AVE prévio, idade avançada, índice de massa corpórea baixa e risco aumentado de sangramento) indica-se o uso de clopidogrel, sendo sugerido a realização de avaliação de atividade plaquetária.^30 , 32^

Remdesivir é um antirretroviral em investigação para COVID-19 e que já foi utilizado durante a epidemia de Ebola. Apesar de medicação promissora, em estudo randomizado, duplo-cego, multicêntrico, a prescrição de remdesivir não resultou em desfechos de mortalidade em relação ao placebo. Houve tendência a diminuição de sintomas, porém sem significância estatística.^33^ Não há descrições sobre cardiotoxicidade e outras interações medicamentosas importantes até o momento.^30 , 32^

### 3.2. Hidroxicloroquina e Cloroquina

Hidroxicloroquina e cloroquina são medicações frequentemente utilizadas em pacientes portadores de malária e outras doenças inflamatórias sistêmicas tais como lúpus eritematoso sistêmico e artrite reumatoide. Aparentemente, são capazes bloquear a entrada de vírus pelas células, além de produzir imunomodulação, atenuando a produção de citocinas, a inibição de autofagia e atividade lisossomal no hospedeiro. Podem exercer propriedades antitrombóticas, especialmente contra anticorpos antifosfolípides.^5 , 34 , 35^

Há evidências anteriores do uso e de sua eficácia em epidemias prévias no tratamento de SARS e MERS. Um estudo chinês demonstrou que em 100 pacientes infectados pelo COVID-19, o uso de cloroquina foi relacionado a melhora do padrão radiológico, maior depuração viral e menor progressão da doença. Apesar dos resultados promissores, o estudo apresenta diversas limitações e tem diversos vieses de interpretação.^5 , 34 , 35^ Em outras publicações de estudos observacionais, demonstrou-se que o uso de hidroxicloroquina associado ou não ao uso de azitromicina não resultou em desfechos favoráveis. Não houve diminuição de mortalidade ou tempo de intubação, assim como não houve diferença de soroconversão em pacientes com doença leve a moderada.^36 - 38^ Diversos estudos sobre o impacto da hidroxicloroquina e/ou cloroquina estão em andamento.

Apesar de classicamente bem toleradas, a cloroquina e a hidroxicloroquina podem induzir graves efeitos colaterais tais como aumentar o intervalo QT, induzir hipoglicemia, retinopatia e distúrbios neuropsiquiátricos. No entanto, não há descrição de interação com medicamentos antiplaquetários ou anticoagulantes.^5 , 34 , 35^

### 3.3. Corticosteroides

Metilprednisolona é outra droga cujo uso pode ser considerado durante a apresentação grave de COVID-19 e síndrome do desconforto respiratório agudo. Sabidamente provoca retenção hídrica, alterações hidroeletrolíticas e hipertensão. Porém, também não há descrição de interação com medicamentos antiplaquetários ou anticoagulantes.^35^

### 3.4. Heparinas

Em estudo chinês^39^ com 449 pacientes internados por COVID-19, mostrou-se que a estratégia de prescrição de enoxaparina 40-60mg/dia ou heparina não-fracionada 10.000 a 15.000U/dia trouxe benefícios de mortalidade em 28 dias em 2 subgrupos. Um deles era de pacientes com critérios de SIC > 4 (que usa os critérios de aumento de TP, queda na contagem de plaquetas e aumento do SOFA-Score), com diferença de 40% vs. 64,2% (p = 0,029) e o outro subgrupo era composto por pacientes com D-dímero > 6x o limite da normalidade, com diferença de 32,8% vs. 52,4% (p = 0,017), demonstrando que a estratégia de prescrição de profilaxia química de TEV ou anticoagulação plena devem ser consideradas individualmente em todos os pacientes com COVID-19 internados, assim como a pesquisa de eventos trombóticos deve ser buscada com maior intensidade.^26 , 27 , 31 , 39 - 41^

### 3.5. Imunoglobulinas e Anticorpos Anti-IL6

A lógica por trás do uso da imunoglobulina depende do uso dos mecanismos: neutralização viral e imunomodulação. Uma aplicação intrigante do mecanismo anterior é o uso de soro convalescente ou plasma. Tal terapia tem efeitos pleiotrópicos que culminam na supressão da inflamação e, portanto, esta terapia pode potencialmente aliviar a gravidade da doença na fase de hiperinflamação. Evidências mais robustas são necessárias para confirmar esses achados. Da mesma forma, há uma boa razão para se perguntar se os pacientes com COVID-19 com tempestade de citocinas podem se beneficiar de anticorpos monoclonais direcionados ao receptor de interleucina 6(IL-6) que foram bem-sucedidos em atenuar a inflamação em pacientes transplantados. Talvez isso se reflita no contexto trombótico do paciente, porém ainda não há evidências concretas sobre isso.^15^

Anticorpos anti-IL 6 aumentam a expressão da CYP3A4. No entanto, não existe nenhuma recomendação de ajuste de dose de antiplaquetários ou anticoagulantes em pacientes em uso dessa terapia.^5^

## 4. Recomendações

### 4.1. Anticoagulantes

A prescrição de heparina de baixo peso molecular ou heparina não-fracionada profiláticas para TEV ou como anticoagulação plena devem ser individualizadas, sendo sempre consideradas em pacientes de alto risco de TEV internados.^5 , 29 , 39 - 43^

A terapia anticoagulante em pacientes com COVID-19 grave e indícios de SIC e/ou com D-dímero muito elevado em associação a outros biomarcadores que denotam gravidade, na ausência de contraindicação à anticoagulação, pode ser considerada uma estratégia terapêutica fundamentada no consenso de especialistas e em poucos estudos retrospectivos. Adicionalmente, essa estratégia requer a utilização de protocolos institucionais rígidos que permitam a vigilância e a rápida intervenção frente a complicações.^29^

**Figura 1:**
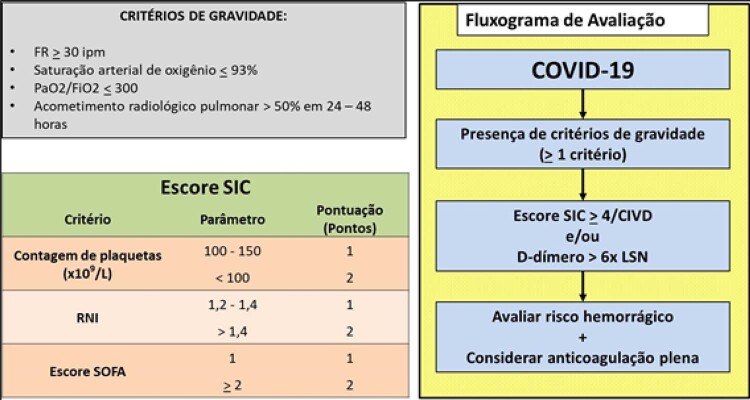
– *Sugestão de manejo e fluxograma para avaliação de terapia anticoagulante em pacientes graves com COVID-19.* FR: frequência respiratória; SIC: coagulopatia induzida pela sepse; RNI: razão de normatização internacional; SOFA: sequential organ failure assessment; CIVD: coagulação intravascular disseminada; LSN: limite superior da normalidade.

É possível que a terapêutica anticoagulante seja mais benéfica quando iniciada na fase pré-trombótica do que nos quadros avançados, quando o risco de sangramento é maior. Em se optando pela anticoagulação, parece razoável o uso de heparina de baixo peso molecular como fármaco de escolha em pacientes estáveis e com depuração normal de creatinina (dose de 1 mg/kg de 12/12h, subcutânea). Em caso de choque ou depuração de creatinina abaixo de 50 ml/min/m^2^ , é preferível o uso de heparina não-fracionada intravenosa (18 UI/kg/h), tendo como alvo um tempo de tromboplastina parcial ativada entre 1,5 e 1,8. Entretanto, não há evidências que fundamentem a ampla utilização de heparina em dose terapêutica na COVID-19. A Figura 1 ilustra uma sugestão de avaliação e estratégia terapêutica nesse grupo de pacientes baseado nas poucas evidências vigentes.^29^

Já a *European Society of Cardiology* propôs uma recomendação de realizar anticoagulação plena em todos os pacientes com sinais de gravidade como: aumento da frequência respiratória > 24 ipm, saturação arterial de oxigênio < 90%, proteína-C reativa elevada, níveis elevados de D-dímero ou em ascensão e níveis de fibrinogênio elevados. Especificamente em relação ao D-dímero a orientação é de anticoagulação plena quando > 3.000 ng/mL, somente profilaxia química quando < 500 ng/mL e uso de enoxaparina 40 mg de 12/12h quando D-dímero estiver entre 500 e 3.000 ng/mL.^42^

**Figura 2:**
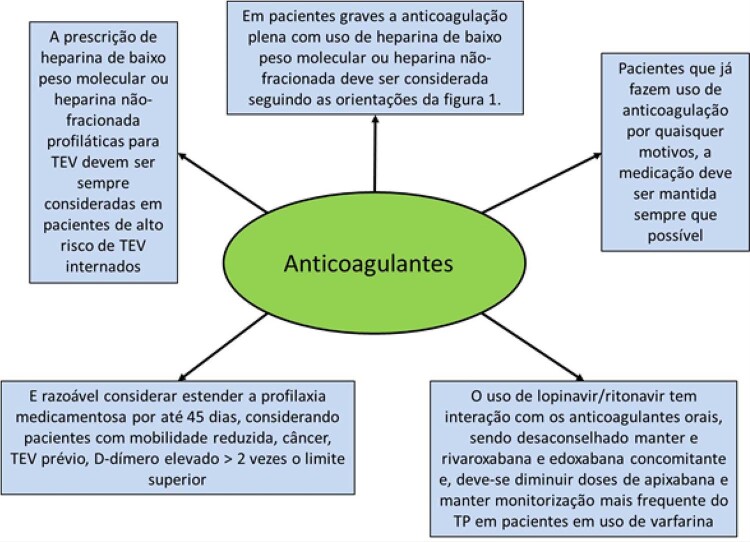
– *Recomendação quanto ao uso de anticoagulantes em pacientes com infecção por COVID-19.* TEV: tromboembolismo venoso; TP: tempo de protrombina.

A manutenção de profilaxia para tromboembolismo após a alta hospitalar também deve ser individualizada com heparina de baixo peso molecular ou novos anticoagulantes, pesando risco benefício de eventos trombóticos *versus* sangramento ( Figura 2 ).

Enquanto não há evidência específica para COVID-19, é razoável considerar individualizar a estratificação de risco de eventos tromboembólicos e hemorrágicos e estender a profilaxia medicamentosa por até 45 dias, considerando pacientes com mobilidade reduzida, câncer, TEV prévio, D-dímero elevado > 2 vezes o limite superior.^5^

Em relação aos pacientes que já fazem uso de anticoagulação prévia por quaisquer motivos, a medicação deve ser mantida sempre que possível. Caso o paciente seja internado por sintomas de pneumonia por COVID-19 a manutenção da medicação deve ser individualizada. Em pacientes graves pode-se ter alterações na farmacocinética das medicações, insuficiência renal, insuficiência hepática, plaquetopneia e CIVD, e a anticoagulação parenteral com uso de heparina de baixo peso molecular ou heparina não-fracionada deve ser preferencial, caso não haja contra-indicações.^5 , 7 , 26 , 27 , 31 , 39 , 40^

O uso de lopinavir/ritonavir tem interação com os anticoagulantes orais, sendo desaconselhado manter e rivaroxabana e edoxabana concomitante e, diminuir doses de apixabana e monitorização mais frequente do TP em pacientes em uso de varfarina. Dessa forma, em pacientes com uso prévio de anticoagulação oral e que necessitam manter a medicação na internação, aconselha-se trocar pela forma parenteral por heparina de baixo peso molecular. É válido lembrar que caso os pacientes evoluam com discrasia sanguínea/CIVD deve-se pesar o risco de sangramento ao manter essas medicações, sendo necessária a suspensão na quase totalidade dos casos.^32^

### 4.2. Antiplaquetários

**Figura 3:**
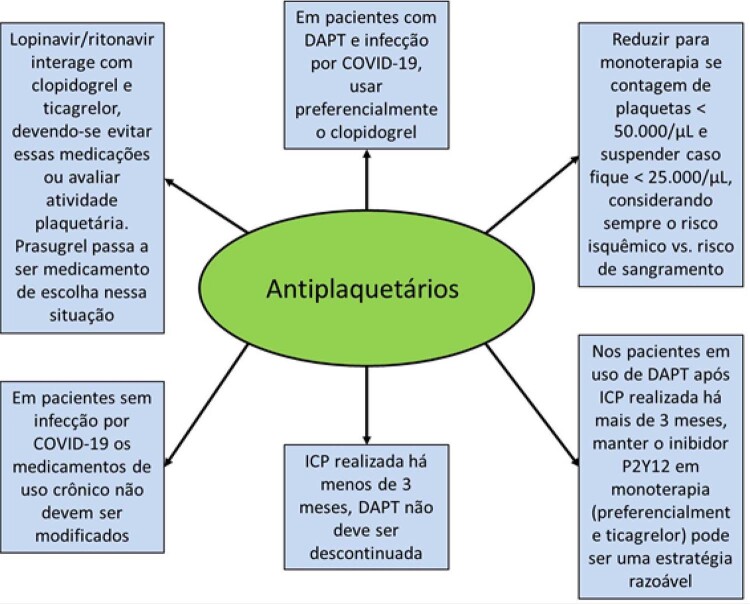
– *Recomendação quanto ao uso de antiplaquetários em pacientes com infecção por COVID-19.* ICP: intervenção coronariana percutânea; DAPT: dupla antiagregação plaquetária.

Em relação ao uso de antiplaquetários, pacientes que fazem uso dessas medicações no cenário de doença coronariana crônica, a medicação deve ser mantida. Em pacientes que usam dupla antiagregação plaquetária, deve-se individualizar a prescrição de tais medicações em pacientes internados ( Figura 3 ).^5 , 7 , 40^

Dado o alto risco de sangramento em pacientes após intervenção coronariana percutânea (ICP) complicada pela COVID-19, a menor duração da dupla antiagregação plaquetária (DAPT) pode ser benéfica nesta população, além do uso preferencial do clopidogrel naqueles com risco de sangramento elevado, pesando-se sempre o risco de trombose de *stent* vs. sangramento. Para contrabalancear o aumento do risco de hemorragia associado à DAPT, estudos mais recentes forneceram evidências que suportam a suspensão precoce de aspirina após ICP, reduzindo principalmente taxas de sangramento. Entre os pacientes em uso de DAPT, manter o inibidor P2Y12 em monoterapia (preferencialmente ticagrelor) pode ser uma estratégia razoável após ICP realizada há mais de 3 meses. Devido à falta de evidências, para aqueles com ICP realizada há menos de 3 meses, DAPT não deve ser descontinuada.^5 , 7^

Caso o paciente necessite do uso de antivirais, há interação entre lopinavir/ritonavir com clopidogrel e ticagrelor, devendo-se evitar essas medicações ou avaliar atividade plaquetária. Prasugrel pode ser administrado com cautela, salvo contra-indicações inerentes ao medicamento. Não há descrição de interações com antiplaquetários endovenosos como cangrelor.^11 , 30 , 32^

Consensos de especialistas recomendam medidas proativas ou mesmo parar toda a terapia antiplaquetária em pacientes com uma contagem de plaquetas < 100.000/μL e < 50.000/μL, respectivamente.^7^ Há entretanto recomendação mais atual que sugere reduzir para monoterapia se < 50.000/μL e suspender caso fique < 25.000/μL, considerando sempre o risco isquêmico vs. risco de sangramento.^5^

### 4.3. Trombolíticos

Tanto a *American Heart Association* quanto a *European Society of Cardiology* indicam o uso de trombólise como primeira opção em pacientes suspeitos/confirmados com COVID-19 e infarto agudo do miocárdio com supradesnivelamento do segmento ST, principalmente em centros sem serviço de hemodinâmica ou naqueles com hemodinâmica disponível, porém sem preparo adequado para evitar a contaminação da equipe envolvida.^5 , 7 , 44 - 47^

Dessa forma, não existe até o momento nenhuma contra-indicação ao uso de trombolíticos nesse contexto e seu uso deve ser pautado de acordo com as contra-indicações usuais, sendo individualizado em situações de instabilidade elétrica ou hemodinâmica, CIVD, plaquetopenia, sangramentos e insuficiência renal ou hepática.^48^

Vale ressaltar que diagnósticos diferenciais de supradesnivelamento do segmento ST devem ser sempre aventados, como miopericardite, nos quais a trombólise deve ser evitada.^5^

## 5. Considerações Finais

As evidências a respeito da COVID-19 e suas interações com os sistemas de coagulação e ativação plaquetária ainda são iniciais. Existem fortes indícios de que essa via possa ser um alvo terapêutico importante. No entanto, ainda são necessários estudos mais robustos para determinar a real importância dos mecanismos pró-trombóticos e a melhor terapia a ser adotada nesse grupo de pacientes.

**Tabela 1:** – Recomendações gerais para sobre Covid-19/antiplaquetários e anticoagulantes

Indicação	Classe de Recomendação	Nível de Evidência
Associação medicamentosa entre terapias antritrombóticas e medicações utilizadas no tratamento do COVID-19		
Em pacientes em uso de Lopinavir-Ritonavir o Prasugrel deve ser o antiplaquetário de escolha	IIB	B
Em pacientes em uso de Lopinavir-Ritonavir, caso contra-indicado Prasugrel, deve-se escolher Clopidogrel	IIB	B
Em pacientes em uso de Lopinavir-Ritonavir, caso optado pelo Clopidogrel, a monitorização de atividade plaquetária	IIB	B
Em pacientes em uso de Lopinavir-Ritonavir, o uso de Ticagrelor deve ser desencorajado	IIB	B
Em pacientes em uso de anticoagulação prévia e que farão uso de Lopinavir-Ritonavir, deve-se trocar o anticoagulante pela forma parenteral (Heparinas)	IIA	B
Em pacientes em uso de anticoagulação prévia, evitar associação de Rivaroxabana ou Edoxabana com Lopinavir-Ritonavir	III	B
Em pacientes em uso de anticoagulação prévia com varfarina, com necessidade de manutenção desta medicação e estiverem em uso de Lopinavir-Ritonavir, deve-se avaliar com maior frequência o TP	IIA	B
O uso de Remdesevir não tem interação medicamentosa importante com antiplaquetários e anticoagulantes	IIA	B
O uso de corticosteróides não tem interação medicamentosa importante com antiplaquetários e anticoagulantes	IIA	B
Imunoglobulinas e anticorpos anti-IL6 não tem interação medicamentosa importante com antiplaquetários e anticoagulantes	IIB	B
O uso de Hidroxicloroquina ou Cloroquina não tem interação medicamentosa importante com antiplaquetários e anticoagulantes	IIB	B
Em pacientes que fizerem uso de Hidroxicloroquina ou Cloroquina, deve-se monitorizar o intervalo QT	IA	B
Uso de anticoagulantes em pacientes infectados pelo COVID-19		
Profilaxia química para eventos tromboembólicos deve ser instituída em todos pacientes internados	IIA	B
A anticoagulação plena deve ser considerada em casos especiais, pesando risco-benefícios, como por exemplo, utilizando o escore de coagulopatia induzida por sepse ou D-Dímero > 6x o limite superior da normalidade	IIB	B
Em pacientes que fazem uso de anticoagulação anteriormente, a medicação deve ser mantida sempre que possível	IIA	B
Considerar estender profilaxia química para eventos tromboembólicos até 45 dias após a alta em pacientes de risco	IIB	B
Uso de antiplaquetários em pacientes infectados pelo COVID-19		
Em pacientes que faziam uso no cenário de doença coronariana crônica, a medicação deve ser mantida	IIA	C
Em pacientes em uso de dupla antiagregação no cenário pós angioplastia (ATC), pode-se considerar manter em monoterapia os pacientes com ATC com duração maior que 3 meses, pesando o risco de sangramento e trombose de stent	IIA	B
