# Supplementary material for: Posicionamento sobre Uso de Antiplaquetários e Anticoagulantes nos Pacientes Infectados pelo Novo Coronavírus (COVID-19) – 2020
Source: Arq Bras Cardiol. 2020 Aug 19;115(2):292–301. [Article in Portuguese] doi: 10.36660/abc.20200424 (PMC8384288; doi:10.36660/abc.20200424)
Supplement: Translation

# Translation: Position Statement on the Use of Antiplatelet Agents and Anticoagulants in Patients Infected with the New Coronavirus (COVID-19) – 2020

**Authors:** Alexandre de Matos Soeiro, Tatiana de Carvalho Andreucci Torres Leal, Marcel de Paula Pereira, Eduardo Gomes Lima, Ana Cristina Baptista da Silva Figueiredo, João Luiz Fernandes Petriz, Dalton Betolim Precoma, Carlos Vicente Serrano

**Affiliations:** 1 Brazil Instituto do Coração (Incor), Hospital das Clínicas da Faculdade de Medicina da Universidade de São Paulo (HCFMUSP), São Paulo, SP – Brazil; 2 Brazil Hospital BP Mirante, São Paulo, SP – Brazil; 3 Brazil Hospital 9 de Julho, São Paulo, SP – Brazil; 4 Brazil Hospital Glória D’Or, Rio de Janeiro, RJ – Brazil; 5 Brazil Hospital Barra D’Or, Rio de Janeiro, RJ – Brazil; 6 Brazil Sociedade Hospitalar Angelina Caron, Curitiba, PR – Brazil; 7 Brazil Pontifícia Universidade Católica do Paraná, Curitiba, PR – Brazil

**Table:** 

Declaration of potential conflict of interests of authors/collaborators of the Position Statement of the Brazilian Society of Cardiology Department of Exercise Testing, Sports Exercise, Nuclear Cardiology, and Cardiovascular Rehabilitation (DERC/SBC) on Activities Within its Scope of Practice During the COVID-19 Pandemic If, within the last 3 years, the author/collaborator of the statement:							
Names of statement collaborators	Participated in clinical and/or experimental studies sponsored by pharmaceutical or equipment companies related to this statement	Spoke at events or activities sponsored by industry related to this statement	Was (is) a member of a board of advisors or a board of directors of a pharmaceutical or equipment industry	Participated in normative committees of scientific research sponsored by industry	Received personal or institutional funding from industry	Wrote scientific papers in journals sponsored by industry	Owns stocks in industry
Antônio Carlos Avanza Jr.	No	No	No	No	No	No	No
Carlos Alberto Cordeiro Hossri	No	No	No	No	No	No	No
Carlos Alberto Cyrillo Sellera	No	No	No	No	No	No	No
Gabriel Blacher Grossman	No	No	No	No	No	No	No
Lara Terra F. Carreira	No	No	No	No	No	No	No
Luiz Eduardo Fonteles Ritt	No	No	No	No	No	No	No
Luiz Eduardo Mastrocola	No	No	No	No	No	No	No
Mauricio Batista Nunes	No	No	No	No	No	No	No
Mauricio Milani	No	No	No	No	No	No	No
Nabil Ghorayeb	No	No	No	No	No	No	No
Odilon Gariglio Alvarenga de Freitas	No	No	No	No	No	No	No
Pedro Ferreira de Albuquerque	No	No	No	No	No	No	No
Romeu Sergio Meneghelo	No	No	No	No	No	No	No
Salvador Manoel Serra	No	No	No	No	No	No	No
Tales de Carvalho	No	No	No	No	No	No	No
William Azem Chalela	No	No	No	No	No	No	No

## 1. Introduction

The new coronavirus (SARS-CoV-2) pandemic has led to debate about the best treatment for the disease and its complications. Recent publications have shown that cardiovascular disease is among the main risk factors for disease progression, including high blood pressure and diabetes mellitus.^1 - 6^

It has been demonstrated that patients infected with the new coronavirus (COVID-19) have distinct activation of prothrombotic mechanisms, including a greater likelihood of thrombotic events. Acute coronary syndrome (ACS) with or without ST-elevation can occur in patients with COVID-19, although the actual incidence is still uncertain.^7 - 10^ Thus, several issues related to using antiplatelet agents and anticoagulants in patients with suspected or confirmed COVID-19 infection remain uncertain. The following recommendations are valid for the most diverse clinical situations, such as atrial fibrillation, acute coronary syndrome, chronic coronary artery disease, percutaneous coronary intervention, post-cardiac surgery, ischemic stroke, and venous thromboembolism, and should be applied on a case-by-case basis.

## 2. Pathophysiology

### 2.1. Mechanism of Cellular Entry

The functional receptor and gateway of the SARS-CoV-2 virus is angiotensin-converting enzyme 2. This carboxypeptidase has the opposite effect of angiotensin-converting enzyme 1, i.e., it increases the degradation of angiotensin 2 and thus has a vasodilating effect. In addition to being present in lung parenchyma, angiotensin-converting enzyme 2 is also distributed throughout the cardiovascular system, kidneys and heart. It is known that angiotensin-converting enzyme 2 has a certain role in ventricular function. Severe left ventricular dysfunction occurred in animal models that reduced the expression of angiotensin-converting enzyme 2. Apparently, infection with the new coronavirus can promote downregulation of these receptors, which could lead to myocardial injury and lung injury.^1 , 2 , 11^ Despite this possible association, an observational study of 8,910 patients infected with SARS-Cov-2 found no increase in mortality among patients using angiotensin-converting enzyme inhibitors or angiotensin receptor blockers.^12^ Thus, these medications should not be discontinued in patients who used them prior to infection.

### 2.2. Myocardial Injury

Several studies, mainly Chinese, have demonstrated the impact of myocardial injury on the progression and severity of COVID-19. Chinese data indicate the presence of myocardial injury in approximately 20 to 30% of hospitalized patients, as well as in 40% of those who died. There are data showing that elevations in some markers, such as troponin and D-dimer, are associated with worse prognosis, intensive care unit hospitalization, mechanical ventilation, and death.^2 , 13 , 14^

The mechanism of myocardial injury is still not well established. In addition to the direct effect of the virus, there is myocardial stress induced by respiratory failure and hypoxemia, including supply and demand imbalances, as well as the indirect action of systemic inflammatory response on myocardial tissue and endothelial function.^14 - 19^

Data on the effects of myocardial inflammation is also insufficient. It is not clear whether COVID-19-induced myocarditis produces heart failure with reduced ejection fraction. There have been anatomopathological findings of lymphocytic myocarditis, even in patients with preserved ejection fraction and signs of ventricular hypertrophy.^14 - 19^

### 2.3. Association with Acute Coronary Syndrome

The increased risk of ACS in patients with COVID-19 can be explained by increased thrombotic activity, evidenced by the frequent elevation of D-dimer and thrombocytopenia. There is also an increase in coronary events in direct association with viral respiratory infection. Factors related to inflammatory activity (e.g., endothelial dysfunction, platelet activation, macrophage activation, liver dysfunction, tissue factor expression and cytokine release) can increase the risk of atherosclerotic plaque instability. More recent studies have also identified high levels of antiphospholipid antibodies in patients with COVID-19, although it is not known whether this is related to disease severity.^5 , 7 - 10 , 15 , 20^

At the beginning of the infection phase, platelet inhibition can reduce the formation of intravascular fibrin and thrombus. Thus, the use of pre- but not post-admission acetylsalicylic acid was found to be associated with a lower risk of respiratory failure and mortality in patients with community-acquired pneumonia. Despite the fact that all P2Y_12_inhibitors reduce platelet and leukocyte aggregates and platelet-derived proinflammatory cytokines, ticagrelor, which is orally administered, is unique in having an additional well-documented target of inhibition: equilibrative nucleoside transporter 1, which contributes to the inhibition of cellular adenosine uptake. Therefore, ticagrelor has more potent anti-inflammatory properties, although it has not been tested in COVID-19.^7^

It should be pointed out that in view of the high incidence of myocardial injury due to the virus and the indirect effects of infection, non-ischemic disease must be considered in an ACS diagnosis, even in the presence of electrocardiographic changes. A case series was published^21^ of 18 patients in New York hospitals with COVID-19 and ST-segment elevation suggestive of acute myocardial infarction, including four with diffuse ST-segment elevation and 14 with focal ST-segment elevation. Of these patients, 50% had coronary angiography and 33% had no obstructive coronary disease. In all, 44% of the patients were diagnosed with acute myocardial infarction. Thus, even in patients with ST-segment elevation, differential diagnoses must be considered due to the heterogeneous clinical picture. However, regardless of the etiology, mortality was 72%. Therapeutic decision making and invasive stratification strategies should consider the clinical condition, the findings of complementary tests, the team’s experience, and the availability of the hemodynamics laboratory.^14 , 16^ In two other studies, a lower incidence of ACS was found in northern California compared to the same period the previous year, while a higher incidence of out-of-hospital cardiac arrest was found in Italy. This suggests a lower demand for emergency services by the population.^22 , 23^

In addition to all these factors, the effects of social isolation have raised enormous concern. Most people have drastically reduced physical activity. In addition, diets can become inadequate due to higher carbohydrate intake. Such lifestyle changes may contribute to thrombotic events, including stroke and ACS.^24^

### 2.4. Thromboembolic Mechanism

Patients infected with COVID-19 are likely to have an increased risk of venous thromboembolism (VTE). Although a large case series has not yet been published, there are reports of abnormal coagulation parameters in hospitalized patients with severe COVID-19.^25 - 27^

A recent case series of 106 COVID-19 patients who underwent pulmonary arteriography found that 30% had VTE. Patients with COVID-19 and pulmonary embolism had higher levels of D-dimer than those without an embolism (p < 0.001), in addition to a greater need for intensive care (75% vs. 32%, p < 0.001). A D-dimer level > 2,660 µg/L showed 100% sensitivity and 67% specificity for pulmonary embolism.^28^

D-dimer levels have been associated with higher mortality rates and seem to progressively increase with infection severity. The phase of the disease in which acute respiratory distress syndrome develops and the radiological pattern worsens is marked by a significant increase in D-dimer levels, which is observed in the most severe cases of myocardial injury and disseminated intravascular coagulation. The systemic inflammatory response in infected patients can result in endothelial injury with a consequent increase in thrombin production and a reduction in endogenous fibrinolysis. This prothrombotic state is called sepsis-induced coagulopathy and precedes disseminated intravascular coagulation. The various mechanisms involved in sepsis-induced coagulopathy act simultaneously, culminating in a prohemostatic state. Apparently, inflammatory cytokines are the most important mediator of this clotting disorder during sepsis.^29^

A cross-interaction has been demonstrated between inflammation and coagulation, with inflammation inducing coagulation activation and coagulation accentuating inflammatory activity. Platelets play a central role in the development of coagulation abnormalities in sepsis and can be activated directly by pro-inflammatory mediators, such as platelet activating factor, as well as through thrombin. Platelet activation can also stimulate fibrin formation by an alternative mechanism. The expression of P-selectin in the platelet membrane not only induces the adhesion of platelets to leukocytes and endothelial cells but also increases the expression of tissue factor in monocytes. Under normal circumstances, coagulation activation is controlled by three important physiological anticoagulant pathways: the antithrombin system, the activated protein C system, and the tissue factor pathway inhibitor. In sepsis, all three pathways become dysfunctional. In the midst of this imbalance in the coagulation system, endogenous fibrinolysis is greatly reduced.^29^

In a Chinese retrospective cohort study, elevated D-dimer levels (> 1 g/L) were strongly associated with hospital death. In another study comparing COVID-19 survivors and non-survivors, non-survivors had significantly higher levels of D-dimer and fibrin degradation products, and 71.4% of non-survivors met the clinical criteria for disseminated intravascular coagulation during the course of disease. In addition to disseminated intravascular coagulation, critically ill patients who have experienced prolonged immobilization are at high risk of VTE. Vascular inflammation can also contribute to a state of hypercoagulation and endothelial dysfunction in such patients. In seriously ill patients with COVID-19 who clinically deteriorate, as evidenced by hypoxia or hemodynamic instability, thromboembolic disease should be considered.^25 - 27^

## 3. Drug Interactions and Cardiovascular Pro- /Antithrombotic Effects

So far, there is no specific treatment for COVID-19 infection, and a variety of therapeutic regimens have been used in severe cases in a hospital environment, although the efficacy and safety of many are still being investigated. Since these drugs can be used in specific situations, it is worth considering their side effects on the cardiovascular system and possible drug interactions with other therapies frequently used in cardiac patients.

### 3.1. Antiretrovirals

Ribavirin and remdesivir block RNA polymerase, while lopinavir-ritonavir inhibits viral replication. Ribavirin-induced cardiotoxicity has not been reported. However, lopinavir-ritonavir prolongs the QT and PR intervals, especially in patients who already have long QT intervals or are using other medications that interact with the QT interval.

Both ribavirin and lopinavir-ritonavir enhance the anticoagulant effect, modifying the action of warfarin (mainly ribavirin) and new anticoagulants such as apixaban and rivaroxaban (mainly lopinavir-ritonavir).^30 - 32^ In another study, an association of dabigatran and antivirals in hospitalized COVID-19 patients resulted in increased in serum plasma levels, requiring medication withdrawal in more than half of the patients.^31^

Lopinavir-ritonavir may also influence the activity of P2Y_12_inhibitors by inhibiting CYP3A4, which reduces the serum level of active metabolites of clopidogrel and increases the activity of ticagrelor. Therefore, due to the high risk of bleeding, the concomitant use of ticagrelor and lopinavir-ritonavir is discouraged.^11^

There is also evidence that using clopidogrel during treatment with lopinavir-ritonavir may result in an insufficient antiplatelet effect, although this has not been observed with prasugrel, which makes it the ideal medication.^32^ In the presence of contraindications for prasugrel (e.g., previous stroke, advanced age, low body mass index, and increased risk of bleeding), clopidogrel is indicated, and platelet activity should be evaluated.^30 , 32^

Remdesivir, an antiretroviral under investigation for use with COVID-19, was used during the Ebola epidemic. Despite its promise, in a randomized, double-blind, multicenter study, it did not result in better mortality outcomes than placebo. Although there was a trend toward symptom reductions, it was not significant.^33^ No cardiotoxicity or other important drug interactions have been reported for this drug.^30 , 32^

### 3.2. Hydroxychloroquine and Chloroquine

Hydroxychloroquine and chloroquine are frequently used in patients with malaria and other systemic inflammatory diseases, such as systemic lupus erythematosus and rheumatoid arthritis. Apparently, they can block viral entry into cells, in addition to stimulating immunomodulation, attenuating the production of cytokines, and inhibiting autophagy and lysosomal activity in the host. They may also have antithrombotic properties, especially against antiphospholipid antibodies.^5 , 34 , 35^

These drugs have been used in previous SARS and MERS epidemics, and there is evidence of their efficacy. A Chinese study of 100 patients with COVID-19 found that chloroquine was associated with radiological improvement, increased viral clearance, and decreased disease progression. Despite its promising results, the study had several limitations and can be interpreted in different ways.^5 , 34 , 35^ In other observational studies, hydroxychloroquine, associated or not with azithromycin, did not result in favorable outcomes. No decrease was found in mortality or intubation time, and no difference in seroconversion was found in patients with mild to moderate disease.^36 - 38^ Several studies are ongoing about the impact of hydroxychloroquine and/or chloroquine.

Although generally well tolerated, chloroquine and hydroxychloroquine can have serious side effects, such as prolonging the QT interval and inducing hypoglycemia, retinopathy and neuropsychiatric disorders. However, no interaction with antiplatelet agents or anticoagulants has been reported.^5 , 34 , 35^

### 3.3. Corticosteroids

Methylprednisolone is another medication that can be considered during severe cases of COVID-19 and acute respiratory distress syndrome. Although it is known to cause water retention, hydrolytic changes and hypertension, interaction with antiplatelets or anticoagulants has not been reported.^35^

### 3.4. Heparins

A Chinese study^39^ of 449 hospitalized COVID-19 patients found that a prescription strategy of enoxaparin 40-60 mg/day or unfractionated heparin 10,000-15,000 U/day resulted in 28-day mortality benefits in two subgroups. In one of these groups, which consisted of patients with criteria for sepsis-induced coagulopathy > 4 (increased prothrombin time, decreased platelet count, and increased SOFA-Score), there was a difference of 40% vs. 64.2% (p = 0.029). The other subgroup, which consisted of patients with > 6 times the normal D-dimer concentration, there was a difference of 32.8% vs. 52.4% (p = 0.017). This demonstrates that chemical prophylaxis for VTE or full anticoagulation therapy should be considered on a case-by-case basis in all hospitalized patients with COVID-19, and a careful search should be made for thrombotic events.^26 , 27 , 31 , 39 - 41^

### 3.5. Immunoglobulins and Anti-IL6 Antibodies

Immunoglobulin use depends on two mechanisms: viral neutralization and immunomodulation. One intriguing application of viral neutralization is convalescent serum or plasma. Since this therapy has pleiotropic effects that result in suppressed inflammation, this therapy can potentially alleviate disease severity in the hyperinflammatory phase. More robust evidence is needed to confirm these findings. Likewise, it may be that COVID-19 patients with a cytokine storm can benefit from monoclonal antibodies targeting interleukin-6 receptor, since it has been successful in mitigating inflammation in transplanted patients. Although this could be reflected in the patient’s thrombotic state, there is still no concrete evidence about it.^15^

Although anti-interleukin-6 receptor antibodies increase CYP3A4 expression, there are no dose-adjustment recommendations regarding patients using antiplatelet agents or anticoagulants.^5^

## 4. Recommendations

### 4.1. Anticoagulants

The decision to prescribe low molecular weight heparin or prophylactic unfractionated heparin for VTE or as full anticoagulation therapy should be made on a case-by-case basis, and should always be considered in hospitalized high-risk VTE patients.^5 , 29 , 39 - 43^

Based on expert consensus and the results of a few retrospective studies, when anticoagulation is not contraindicated, anticoagulant therapy can be considered for use in patients with severe COVID-19 and signs of sepsis-induced coagulopathy and/or very high D-dimer levels in association with other biomarkers that denote severity. This strategy requires the use of strict institutional protocols that allow for surveillance and rapid intervention when complications arise.^29^

It is possible that anticoagulant therapy is more beneficial when initiated in the pre-thrombotic phase than in advanced cases, when the risk of bleeding is greater. When opting for anticoagulation, it would seem reasonable to use low molecular weight heparin as the drug of choice in stable patients with normal creatinine clearance (subcutaneous dose of 1 mg/kg every 12 hours). In case of shock or creatinine clearance below 50 mL/min/m^2^, unfractionated intravenous heparin (18 IU/kg/h) is preferable, targeting an activated partial thromboplastin time between 1.5 and 1.8. However, there is no evidence to support the wide use of heparin in therapeutic doses for COVID-19. Figure 1 illustrates a suggested assessment and therapeutic strategy for this group of patients based on current evidence.^29^

**Figure 1:** – *Management suggestion and flowchart for assessing anticoagulant therapy in critically ill patients with COVID-19.* RR: respiratory rate; SIC: sepsis-induced coagulopathy; INR: international normalized ratio; SOFA: sequential organ failure assessment; DIC: disseminated intravascular coagulation; ULN: upper limit of normal.

The European Society of Cardiology recommended full anticoagulation in all patients with signs of severity, e.g., respiratory rate > 24 bpm, arterial oxygen saturation < 90%, high C-reactive protein, high or rising D-dimer levels, and high fibrinogen levels. With respect to D-dimer levels, the recommendation is full anticoagulation when > 3,000 ng/mL, chemical prophylaxis alone when < 500 ng/mL, and 40 mg enoxaparin every 12 hours when between 500 and 3,000 ng/mL.^42^

Prophylaxis for thromboembolism, which should be continued after hospital discharge, must also be individualized with either low molecular weight heparin or new anticoagulants, assessing the risk/benefit of thrombotic events vs. bleeding ( Figure 2 ).

**Figure 2:** – *Recommendations regarding the use of anticoagulants in patients with COVID-19.* VTE: venous thromboembolism; TP: prothrombin time.

Although there is no specific evidence regarding COVID-19, it is reasonable to consider individualizing the risk stratification of thromboembolic and hemorrhagic events and extending drug prophylaxis for up to 45 days in patients with reduced mobility, cancer, previous VTE, high D-dimer levels (i.e., > 2 times the upper limit of normal).^5^

Regarding patients who have been using anticoagulants for any reason, the medication should be continued whenever possible. However, if the patient is hospitalized for COVID-19-associated pneumonia, continuation must be determined on a case-by-case basis. Since there may be changes in the pharmacokinetics of medications, renal failure, liver failure, thrombocytopenia, and disseminated intravascular coagulation in critically ill patients, parenteral anticoagulation with low molecular weight heparin or unfractionated heparin is preferable if there are no contraindications.^5 , 7 , 26 , 27 , 31 , 39 , 40^

There is an interaction between lopinavir-ritonavir and oral anticoagulants. It is not advisable to administer rivaroxaban and edoxaban concomitantly. In patients using warfarin, apixaban doses should be reduced and there should more frequent monitoring of prothrombin time. Thus, in patients who have been using oral anticoagulants and must continue using them after admission, it is advisable to administer low molecular weight heparin in the parenteral form. It should be remembered that if patients evolve to blood dyscrasia or disseminated intravascular coagulation, the risk of bleeding must be considered when continuing these medications, since they are suspended in almost all cases.^32^

### 4.2. Antiplatelet Agents

Patients who use antiplatelet agents for chronic coronary disease should continue taking them. In hospitalized patients on dual antiplatelet therapy, the prescription should be individualized ( Figure 3 ).^5 , 7 , 40^

**Figure 3:** – *Recommendations regarding the use of antiplatelet agents in patients with COVID-19.* PCI: percutaneous coronary intervention; DAPT: double antiplatelet therapy.

Given the high risk of bleeding in patients after percutaneous coronary intervention (PCI) complicated by COVID-19, a shorter duration of dual antiplatelet therapy (DAPT) may be beneficial in this population in addition to the preferential use of clopidogrel in those at high risk of bleeding, although the risk of stent thrombosis vs. bleeding should always be considered. To counterbalance the increased risk of bleeding associated with DAPT, more recent studies have provided evidence in favor of early withdrawal of acetylsalicylic acid after PCI, which mainly reduces bleeding rates. Among patients on DAPT, continuing the P2Y_12_inhibitor in monotherapy (preferably ticagrelor) may be a reasonable strategy when a PCI was performed more than 3 months ago. Due to a lack of evidence, DAPT should not be discontinued for those who received a PCI less than 3 months ago.^5 , 7^

If the patient requires antivirals, there is an interaction between lopinavir-ritonavir and clopidogrel and ticagrelor, and these medications should be avoided or platelet activity evaluated. Prasugrel can be administered with caution, unless there are contraindications. Interactions with intravenous antiplatelet agents such as cangrelor have not been reported.^11 , 30 , 32^

Expert consensus recommends taking proactive measures or even discontinuing all antiplatelet therapy in patients with platelet counts < 100,000/μL and < 50,000/μL, respectively.^7^ There is, however, a more current recommendation that suggests switching to monotherapy if the count is < 50,000/μL and suspending therapy if the count is < 25,000/μL, always considering ischemic risk vs. bleeding risk.^5^

### 4.3. Thrombolytics

Both the American Heart Association and the European Society of Cardiology indicate the use of thrombolysis as the first option in suspected/confirmed COVID-19 patients with acute myocardial infarction and ST-segment elevation, both in centers without hemodynamic services and in those with hemodynamic services that have not made adequate preparations to avoid contaminating the team.^5 , 7 , 44 - 47^

So far, there is no contraindication for using thrombolytics in this context, and their use should be based on the usual contraindications and should be individualized in situations of electrical or hemodynamic instability, disseminated intravascular coagulation, thrombocytopenia, bleeding and renal or hepatic failure.^48^

It should be pointed out that differential diagnoses of ST-segment elevation should always be considered, such as myopericarditis, for which thrombolysis should be avoided.^5^

## 5. Final Considerations

Evidence about the interaction of COVID-19 with coagulation and platelet activation systems is still early. There is strong evidence that this pathway could be an important therapeutic target. However, more robust studies are still needed to determine the real importance of prothrombotic mechanisms and the best therapy for this group of patients.

**Table 1:** – General recommendations about COVID-19/antiplatelet agents and anticoagulants

Indication	Class of recommendation	Level of evidence
Drug association between antithrombotic therapies and medications used in the treatment of COVID-19		
In patients using lopinavir-ritonavir, prasugrel should be the antiplatelet agent of choice	IIB	B
In patients using lopinavir-ritonavir, if prasugrel is contraindicated, clopidogrel should be used	IIB	B
In patients using lopinavir-ritonavir, if clopidogrel is used, platelet activity should be monitored	IIB	B
Ticagrelor should be discouraged in patients using lopinavir-ritonavir	IIB	B
In patients who are already using anticoagulants and will be using lopinavir-ritonavir, the anticoagulant must be replaced with the parenteral form (heparins)	IIA	B
In patients who are already using anticoagulants, avoid associating rivaroxaban or edoxaban with lopinavir-ritonavir	III	B
In patients who are already using anticoagulants with warfarin (and must continue this medication) and lopinavir-ritonavir, prothrombin time should be evaluated more frequently.	IIA	B
Remdesivir has no significant drug interaction with antiplatelet agents and anticoagulants	IIA	B
Corticosteroids have no significant drug interaction with antiplatelet agents and anticoagulants	IIA	B
Immunoglobulins and anti-interleukin-6 antibodies have no significant drug interaction with antiplatelet agents and anticoagulants	IIB	B
Hydroxychloroquine and chloroquine have no significant drug interaction with antiplatelet agents and anticoagulants	IIB	B
In patients using hydroxychloroquine or chloroquine, the QT interval should be monitored	IA	B
The use of anticoagulants in patients with COVID-19		
Chemical prophylaxis for thromboembolic events should be performed for all inpatients	IIA	B
Full anticoagulation should be considered in special cases after assessing the risk/benefits, e.g., using the sepsis-induced coagulopathy score or D-dimer >6 normal	IIB	B
In patients who are already using anticoagulants, the medication should be continued whenever possible	IIA	B
Consider extending chemical prophylaxis for thromboembolic events up to 45 days after discharge for at-risk patients	IIB	B
The use of antiplatelet agents in patients with COVID-19		
The medication patients with chronic coronary disease should be continued	IIA	C
In patients using dual antiplatelet therapy post-angioplasty (with a PCI more than 3 months ago) monotherapy may be considered, as long as the risk of bleeding and stent thrombosis is considered.	IIA	B
